# Let-7f miRNA regulates SDF-1α- and hypoxia-promoted migration of mesenchymal stem cells and attenuates mammary tumor growth upon exosomal release

**DOI:** 10.1038/s41419-021-03789-3

**Published:** 2021-05-20

**Authors:** Virginia Egea, Kai Kessenbrock, Devon Lawson, Alexander Bartelt, Christian Weber, Christian Ries

**Affiliations:** 1 Institute for Cardiovascular Prevention (IPEK), Ludwig-Maximilians-University of Munich, Munich, Germany; 2 Department of Anatomy, University of California, San Francisco, CA USA; 3 German Center for Cardiovascular Research (DZHK), Partner Site Munich Heart Alliance, Munich, Germany; 4 Institute for Diabetes and Cancer (IDC), Helmholtz Center Munich, Neuherberg, Germany; 5 Department of Molecular Metabolism, 665 Huntington Avenue, Harvard T.H. Chan School of Public Health, 02115 Boston, MA USA; 6 Dept. Biochemistry, Cardiovascular Research Institute Maastricht, University of Maastricht, Maastricht, The Netherlands; 7 Munich Cluster for Systems Neurology (SyNergy), Munich, Germany

**Keywords:** Mesenchymal migration, Preclinical research

## Abstract

Bone marrow-derived human mesenchymal stem cells (hMSCs) are recruited to damaged or inflamed tissues where they contribute to tissue repair. This multi-step process involves chemokine-directed invasion of hMSCs and on-site release of factors that influence target cells or tumor tissues. However, the underlying molecular mechanisms are largely unclear. Previously, we described that microRNA let-7f controls hMSC differentiation. Here, we investigated the role of let-7f in chemotactic invasion and paracrine anti-tumor effects. Incubation with stromal cell-derived factor-1α (SDF-1α) or inflammatory cytokines upregulated let-7f expression in hMSCs. Transfection of hMSCs with let-7f mimics enhanced CXCR4-dependent invasion by augmentation of pericellular proteolysis and release of matrix metalloproteinase-9. Hypoxia-induced stabilization of the hypoxia-inducible factor 1 alpha in hMSCs promoted cell invasion via let-7f and activation of autophagy. Dependent on its endogenous level, let-7f facilitated hMSC motility and invasion through regulation of the autophagic flux in these cells. In addition, secreted let-7f encapsulated in exosomes was increased upon upregulation of endogenous let-7f by treatment of the cells with SDF-1α, hypoxia, or induction of autophagy. In recipient 4T1 tumor cells, hMSC-derived exosomal let-7f attenuated proliferation and invasion. Moreover, implantation of 3D spheroids composed of hMSCs and 4T1 cells into a breast cancer mouse model demonstrated that hMSCs overexpressing let-7f inhibited tumor growth in vivo. Our findings provide evidence that let-7f is pivotal in the regulation of hMSC invasion in response to inflammation and hypoxia, suggesting that exosomal let-7f exhibits paracrine anti-tumor effects.

## Introduction

Human mesenchymal stem cells (hMSCs) are in the focus of intensive efforts worldwide, directed not only at elucidating their nature and unique properties, but also directed at developing cell- and secretome-based therapies for a diverse range of diseases. hMSCs were first isolated from bone marrow^1^. Presently, it is known that hMSCs exist in all tissues. In response to chemotactic signals such as SDF-1α and its receptor CXCR4, hMSCs are able to invade through barriers of extracellular matrix (ECM) facilitated by the secretion of matrix metalloproteinases (MMPs)^2,3^. Essentially, hMSCs are characterized by their ability for self-renewal and differentiation into osteocytes, adipocytes, and chondrocytes^4,5^. However, they also give rise to multiple additional cell types such as cardiomyocytes and endothelial-like cells^6,7^. Another peculiarity of hMSCs is their tumor tropism. Attracted by the inflammatory milieu, hMSCs infiltrate neoplastic tissues and affect tumor growth and progression^3,8^. In light of these unique features, hMSCs hold great promise for regenerative medicine and cell-based therapy of various diseases such as myocardial infarction and cancer^9,10^. Evidence accumulates that the diverse physiological roles of hMSCs largely stem from paracrine effects mediated by the release of soluble biologically active factors, the so-called secretome^11^. This includes cytokines, chemokines, growth factors, and regulatory RNA molecules, many of them encapsulated in extracellular vesicles such as exosomes, influencing surrounding cells to decrease inflammation and improve tissue repair^12–14^.

Reduced oxygen concentration (hypoxia) is a major stressor for cells and a prominent feature of the pathological states associated with tissue inflammation and cancer. A central regulator of the cellular response to hypoxia is hypoxia-inducible-factor-1-alpha (HIF-1α). When oxygen is sufficient (normoxia), HIF-1α is continuously produced and degraded, which is controlled by the oxygen-dependent activity of prolyl hydroxylase domain-containing proteins (PHDs). When oxygen is low, HIF-1α is stabilized and activates the expression of genes involved in cell survival and invasion^15^. Hypoxia preconditioning of hMSCs is a novel strategy to improve their survival and therapeutical effects^16,17^. However, the underlying mechanisms and the impact of hypoxia on hMSC functionality and invasive properties remain poorly understood.

Hypoxia/HIF-1α signaling is known to activate the autophagic pathway^18^. Macroautophagy (hereafter autophagy) is a catabolic process used by eukaryotic cells for adapting to stress, maintaining nutrient homeostasis, and mediating quality control by lysosomal degradation of damaged proteins and organelles^19^. Autophagy is facilitated by several autophagy-related gene (ATG) proteins and inhibited by the membrane-associated E3 ubiquitin-protein ligase RNF5^20^. Upon activation of the autophagic pathway, cytosolic LC3-I is lipidated to form LC3-II which is specifically recruited to the surface of autophagosomes^19^. The extent of degradative activity by autophagy, called autophagic flux, impacts proliferation and differentiation of hMSCs^21^. However, the role of autophagy in hMSC invasion is unclear.

Basically, all regulatory networks converge on the regulation of gene expression. MicroRNAs (miRNAs) are small non-coding RNA molecules that play important regulatory roles in multiple biological processes^22^. miRNAs control gene expression posttranscriptionally by binding to their target mRNAs, which leads to the degradation of the mRNA or suppression of its translation^23^. Let-7f belongs to a miRNA family of 12 members and has been demonstrated to control survival and differentiation of hMSCs^24,25^. However, little is known about the importance of let-7f in hMSC invasion and any potential paracrine effects of let-7f on tumor cells.

We have previously demonstrated the role of let-7f in osteogenic differentiation of hMSCs^25^. In the present study, we provide evidence that let-7f is a key regulator of hMSC invasion. SDF-1α, inflammatory cytokines, and hypoxia augmented endogenous let-7f that promoted hMSC invasion via elevated autophagic flux in these cells. Furthermore, overexpression of let-7f in hMSCs facilitated its exosomal release and uptake by mammary tumor cells resulting in reduced tumor growth.

## Materials and methods

### Design of the study

The main objective of our study was to understand the role of let-7f in hMSC invasion and crosstalk with tumor cells. We studied the influence of inflammatory, hypoxic, and autophagic conditions on let-7f expression and the ability of hMSCs to transmigrate through barriers of human ECM enabled by extracellular proteolysis. Experiments in hMSCs involved the application of the chemokine SDF-1α, inflammatory cytokines (i.e., TGF-β, TNF-α, IL-1β), stabilization of HIF-1α (i.e., 1% O_2_, PHD-2 inhibitor IOX2), manipulation of autophagic flux by chemical inducers and inhibitors of autophagy (i.e., rapamycin, 3-methyladenine), an inhibitor of exosome release (i.e., GW4869), or gene manipulation (i.e., let-7f overexpression and inhibition, siRNAs against ATG7) for (1) transcriptional analysis and tracking of let-7f (i.e., qRT-PCR, fluorescently tagged miRNAs, immunocytochemistry, flow cytometry), (2) analysis of downstream effects (i.e., immunoblotting and zymography for relevant mediators), and (3) assessment of cell functions (proliferation, chemotactic invasion, cell motility, release of exosomes). Finally, the pathophysiological significance of hMSC-derived let-7f on tumor growth was tested in a tumor mouse model (i.e., implantation of 3D spheroids with a mixed composition of hMSCs and 4T1 mammary carcinoma cells) studying the relevance of hMSCs in anti-tumor cell therapy.

### Cultivation and treatment of hMSCs

Bone-marrow-derived hMSCs were purchased from Lonza as passage-one cells. Each lot was tested by Lonza for purity using flow cytometry and differentiation capacity into osteogenic, chondrogenic, and adipogenic lineages. The cells were positive for CD29, CD44, CD105, and CD166, and negative for CD14, CD34, and CD45. hMSCs were cultured as described previously^25^, using the StemMACS expansion media (Miltenyi Biotec, Bergisch Gladbach, Germany). For experiments under serum-free conditions, hMSCs were washed with serum-free medium (SFM) and incubated in Dulbecco Modified Eagle Medium (DMEM) (PAA Laboratories, Coelbe, Germany) supplemented with 1% Nutridoma SP (Roche Applied Science, Mannheim, Germany) in the absence or presence of TGF-β at 100 ng/ml, IL-1β at 50 ng/ml, TNF-α at 50 ng/ml, and SDF-1α at 100 ng/ml (all purchased from PeproTech, Rocky Hill, NJ).

For experiments under normoxic conditions, cells were maintained at 37 °C in a humidified air atmosphere in the presence of 21% O_2_ and 5% CO_2_. For experiments under hypoxic conditions, cells were cultured in a Heracell 150i CO_2_ (Thermo Scientific, Schwerte, Germany) at 37 °C in the presence of 1% O_2_ and 5% CO_2_. All experiments were carried out with hMSCs between the fourth and eighth passage which exhibited an average cell doubling time of 96 hours. Cells were tested by us for their ability to differentiate into the adipogenic and osteogenic lineage as described^25^.

For studies on autophagy, cells were grown under serum-free conditions in the absence or presence of 5 µM rapamycin (Santa Cruz Biotechnology), 5 mM 3-methyladenine (3-MA, Sigma Aldrich), or 10 µM bafilomycin A1 (Sigma Aldrich) as described^26^. Cells were routinely tested for vitality and mycoplasma contamination.

### Assessment of cell vitality and proliferation

Cell vitality and proliferation were determined by the use of the WST-8 assay (Dojindo, Rockville, MD, USA) that is based on the cleavage of the tetrazolium salt WST-8 by mitochondrial dehydrogenases in viable cells following the manufacturer’s instructions.

### qRT-PCR analysis of mRNA and miRNA expression

Isolation of total RNA from hMSCs was accomplished using the RNeasy Mini Kit (Qiagen, Hilden, Germany), and on-column DNase digestion with the RNase-free DNase-set (Qiagen) was performed according to the manufacturer’s protocols. The cDNA synthesis was completed following the instructions of the First Strand cDNA Synthesis Kit for RT-PCR (AMV) (Roche Applied Science) using oligo dT primers.

qRT-PCR was carried out on a LightCycler (Roche Applied Science) using LightCycler-FastStart DNA Master SYBR Green I Kit (Roche Applied Science glyceraldehyde-3-phosphate dehydrogenase (GAPDH) as a housekeeping gene standard) according to a previously published protocol^2^. PCR primer sets and kits were applied as listed in Table S1.

miRNA expression was determined using the miScript PCR System (Qiagen) for conversion of RNA into cDNA and SYBR Green-based qRT-PCR for detection. Relative expression was normalized to snord61, snord72, or U2 as single or multiple reference genes and scaled to the sample with the lowest expression. The respective primer sequences and catalogue numbers are provided in Table S1.

### Isolation of exosomes and quantification of let-7f in culture supernatants

Conditioned media was centrifuged at 800 × *g* for 15 min at 4 °C and 100 µL of the media was applied for isolation of vesicular RNA by using the Plasma Exosome and Free-Circulating RNA Isolation Mini kit (Norgen Biotek, Thorold, Canada) following the manufacturer’s instructions. Levels of let-7f were measured by qRT-PCR on undiluted cDNA applying the miScript technology and normalized on the level of the spike-in cel-miR-39 (Snord61/72).

### Transfection of cells with siRNA, miRNA mimics, and miRNA Inhibitors

Specific knockdowns of HIF-1α and ATG7 in hMSCs were accomplished by the application of RNA interference (RNAi) technology. siRNA against HIF1-α, ATG-7, and a non-specific siRNA with no target in the human transcriptome (used as a negative control) were purchased from Qiagen and are listed in Table S1. For studies of miRNA function and gene regulation, miScript miRNA mimics and miScript miRNA inhibitors of let-7f as well as non-specific siRNA control oligonucleotides were applied (Qiagen). The sequences of all siRNAs, miRNA mimics, and miRNA inhibitors used are listed in Table S1. Cells were transfected with 20 nM of siRNAs or miRNAs by use of Lipofectamin 2000 (Invitrogen) as described previously^25^. Fluorescence labeling of let-7f mimics was accomplished by use of the *Label*IT® miRNA labeling kit Cy5 following the protocol provided by the manufacturer (Mirus Bio, Wisconsin, USA). Cell transfection efficiencies were calculated from the number of cells displaying let-7f-Cy5 uptake to the total number of cells visible in the section subjected to fluorescence microscopy analysis with values typically averaging ~80% (Fig. S1).

### Protein extraction, subcellular fractioning, and Western blot analysis

hMSC whole protein extraction was accomplished with a buffer containing 40 mM Tris–HCl pH 8.0, 150 mM NaCl, 1% NP-40, 0.5% sodium deoxycholate and 0.1% SDS supplemented with a mixture of proteinase and phosphatase inhibitors (cOmplete Mini Tablets and PhosSTOP; Roche)^27^.

To obtain nuclear and cytosolic protein extracts, cells were lysed with a buffer containing 10 mM HEPES, 1.5 mM MgCl_2_, 10 mM KCl, 0.5 mM DTT, and 0.05% of EDTA for 15 min on ice. The cell lysate was centrifuged at 3,000 rpm at 4 °C for 10 min. The supernatant was collected (cytoplasm) whereas the pellet was resuspended with a second buffer made of 5 mM HEPES, 1.5 mM MgCl_2_, 0.2 mM EDTA, 0.5 mM DTT, 26% glycerol, and 300 mM NaCl, vortexed, sonicated, and centrifuged at 30,000 × *g* and 4 °C for 30 min. All buffers were supplemented with proteinase and phosphatase inhibitors (cOmplete Mini Tablets and PhosSTOP; Roche). Fifteen to twenty µg of lysates were resolved by SDS/polyacrylamide gel electrophoresis and then transferred to polyvinylidene difluoride membranes as described^2^. The blotted membranes were incubated overnight with the primary antibodies enlisted in Table S2 and then with HRP-conjugated secondary antibodies (Cell Signaling Technology). Bound antibodies were detected using the enhanced chemiluminescence system (GE Healthcare Life Sciences). Recombinant protein standards were used for molecular mass determination. Densitometric quantification was performed using a GS-800 Calibrated Densitometer driven by ImageMaster-1D Elite quantification software (GE Healthcare Life Sciences, Freiburg, Germany) as recommended by the distributor.

### Zymography analysis

Zymography analysis was carried out as described previously^28^. Briefly, samples were run under non-reducing conditions without prior boiling in precast 10% polyacrylamide mini-gels containing 0.1% gelatin as substrate (Invitrogen). After electrophoresis, gels were washed twice for 15 min in 2.7% Triton X-100 on a rotary shaker to remove SDS and to allow proteins to renature. The gels were then incubated in a buffer containing 50 mM Tris–HCl pH 7.5, 200 mM NaCl, 5 mM CaCl_2_, and 0.2% Brij35 (Invitrogen) for 18 h at 37 °C. The zymograms were stained for 90 min with 0.02% Coomassie Blue R-350 in a 30% methanol/10% acetic acid solution using PhastGel-Blue-R tablets (GE Healthcare). Areas of substrate digestion appear as clear bands against a darkly stained background. Densitometric quantification of developed zymograms was performed as described for Western blots. As a marker for the electrophoretic mobility of gelatinases in zymograms we used a conditioned medium from HT1080 fibrosarcoma cells containing MMP-9 and MMP-2^29^.

### Cell invasion assay

Studies on chemotactic invasion of hMSCs were performed using the Costar Transwell chamber system (24-well; Costar, Pleasanta, CA) as previously described in detail^2^. Briefly, membrane filters with a pore size of 8 μm (Costar) were coated with 10 μg human ECM (BD Biosciences, Bedford, MA) which is mainly composed of laminin, collagen type IV, and proteoglycans, providing a composition similar to that of human basement membranes. hMSCs (5 × 10^3^) were placed into the upper compartment of the invasion chamber. The lower compartment of the Transwell system contained 10% human serum (PAA Laboratories) or cytokines/chemokines at the indicated concentrations diluted in SFM as a source of chemoattractants. Each invasion experiment was performed in triplicate. After 48 h of incubation, cells that had migrated into the lower compartment were counted. The invasion rate was calculated from the number of migrated cells to the total cell number.

For inhibition experiments, hMSCs were pre-incubated for 30 min without or with 10 µg/mL AMD3100 (Sigma), a highly specific antagonist for binding of SDF-1 to CXCR4 or 10 µg/mL Ro 206-0222, a highly specific inhibitor for MMP-2, MMP-9, and MMP-14^30^ (kindly provided by Dr. Krell, Roche Diagnostics, Pharma Research Penzberg, Germany). Thereafter, the cells were transferred into the upper compartment of the Transwell system. The respective inhibitors were also added at the same concentrations to the medium in the upper and lower compartment. Preceding measurements had shown that incubation of hMSCs with the inhibitors (10 µg/mL, 48 h) did not significantly affect cell viability and proliferation.

### Quantification of pericellular proteolysis

For quantitative analysis of pericellular proteolytic activity, the QCM^TM^ Gelatin Invadopodia Assay (Millipore) was applied according to the manufacturer’s instructions. Briefly, 10^4^ hMSCs were plated onto a thin uniform layer of fluorescein-labeled gelatin (green) affixed to the bottom of glass chamber 8-well slides (Ibidi, Martinsried, Germany). After incubation for 24 h in a cell incubator at 37 °C and 5% CO_2_, degraded areas of gelatin, now devoid of fluorescence, were visualized by fluorescence microscopy. TRITC-labeled phalloidin (red) and 4′,6-diamidino-2-phenylindole (DAPI) (blue) were used for counterstaining of cytoskeletal F-actin and nuclei, respectively. Areas covered by cells and areas of degraded gelatin were quantified using ImageJ analysis software.

### µ-Slide Chemotaxis^3D^ assay and life cell imaging analysis

Studies on directed migration of hMSCs were performed using the µ-Slide Chemotaxis^3D^ assay (Ibidi, Martinsried, Germany) as previously described by us in detail^27^. Briefly, 0.5 × 10^6^ hMSCs suspended in SFM were placed into the observation channel of a µ-Slide. One medium reservoir was filled with 65 µL RPMI (GE Healthcare) supplemented with 100 ng/mL SDF-1α (PeproTech) and the second reservoir with RPMI only. The µ-Slide was then incubated for 24 h at 37 °C in an IX70 microscope (Olympus, Tokyo, Japan) that was connected to a SensiCam camera (PCO Imaging, Kelheim, Germany). Serial images of cell movement from a total of 30 hMSCs in each observation channel were captured by taking pictures every 20 min. The resulting images were converted to a stack using the ImageJ software (NIH). To determine the migration path of hMSCs, the image stacks were analyzed by deploying the Manual Tracking plug-in as well as the Chemotaxis and Migration Tool (both from Ibidi) following the recommendations of the provider. To quantify and characterize effects on both chemotactic and migratory potential of hMSCs, the Rayleigh test, the moving direction, and the forward migration index (FMI) were determined. The migration distance displays the summary of all cell center movements between images.

### Immunofluorescence microscopy analysis

hMSCs were seeded in glass-bottom chamber eight-well slides (Ibidi) 24 h before experimental treatments. Upon completion of treatments, cells were either fixed and permeabilized in 100% methanol for 10 min at −20 °C (for LC3 staining) or fixed with 2% paraformaldehyde (PFA) for 30 min and permeabilized with 0.1% Triton X-100 in PBS/BSA 1% for 30 min at room temperature. The slides were subsequently incubated overnight at 4 °C with primary antibody against HIF-1α (Table S2). Non-specific isotype antibodies were used as negative controls. Species-specific fluorescently conjugated secondary antibodies were applied for 2 h at room temperature (Table S2). The slides were embedded in Prolong® Diamond antifade mountant (ThermoFisher Scientific) with added 4′,6-diamidino-2-phenylindole (DAPI) to counterstain nuclei. Analysis was performed at 20 °C using the Olympus IX70 microscope (Olympus Europa GmbH, Hamburg, Germany) with objective lens Olympus × 20 (numerical aperture 0.55), and images were taken using SensiCam camera (PCO Imaging, Kelheim, Germany) with Image-ProPlus software (Media Cybernetics, Bethesda, MD, USA).

### 3D spheroid cultures

3D spheroids were generated from cell suspensions of 4T1 cells or 1:1 mixtures of 4T1 cells and hMSCs. For this, 96-well round-bottom Ultra-Low Attachment (ULA) plates (Corning, New York, USA) were used that feature a hydrogel layer which inhibits cellular attachment. A total of 100 × 10^3^ cells/well were seeded in 30 μl of complete medium in ULA plates and maintained at 37 °C in humidified 5% CO_2_ for 2 days. The resulting 3D spheroids were examined by microscopy before application in the mouse tumor model. For fluorescence staining of cells, 4T1 cells and hMSCs were incubated with CellTracker Green CMFDA and CellTracker Red CMTPX, respectively, (both Thermo Fisher Scientific) following the manufacturer’s recommendations before generation of 3D spheroids. Three weeks after spheroid transplantation, live cells were determined in the tissue by intravital fluorescence microscopy analysis.

### Mouse 4T1 breast tumor model

All animal protocols were reviewed and approved by the UCSF IACUC. Mice were maintained under pathogen-free conditions in the UCSF barrier facility. The mouse 4T1 breast tumor model was used as described^31^ with some modifications. Briefly, BALB/c mice (Simonsen Laboratories, Inc.) were divided into four groups at random with four mice per group followed by transplantation of 3D spheroids consisting of 4T1, 4T1/hMSC-C, 4T1/hMSC-M or 4T1/hMSC-I into cleared mammary fat pads of the mice. The spheroids were allowed to grow for 3 weeks before the resulting tumors were removed from the mice and subjected to weight determination by weighing.

### Statistical analysis

Statistics were calculated using Prism 8.1.2 (GraphPad Inc.) and SPSS Statistics version 20.0 (IBM Corporation, Armonk, NY, USA). Values are reported as mean value and standard deviation (SD). Means were calculated from independently performed experiments. Comparisons between two groups were evaluated by a two-sample *t* test. For three or more groups, standard one-way or two-way ANOVA followed by Bonferroni post hoc test were performed. A two-tailed probability value < 0.05 was deemed as statistically significant. No blinding or randomization of groups was applied.

## Results

### Effect of cytokines/chemokines on let-7f in hMSC invasion

**Fig. 1:**
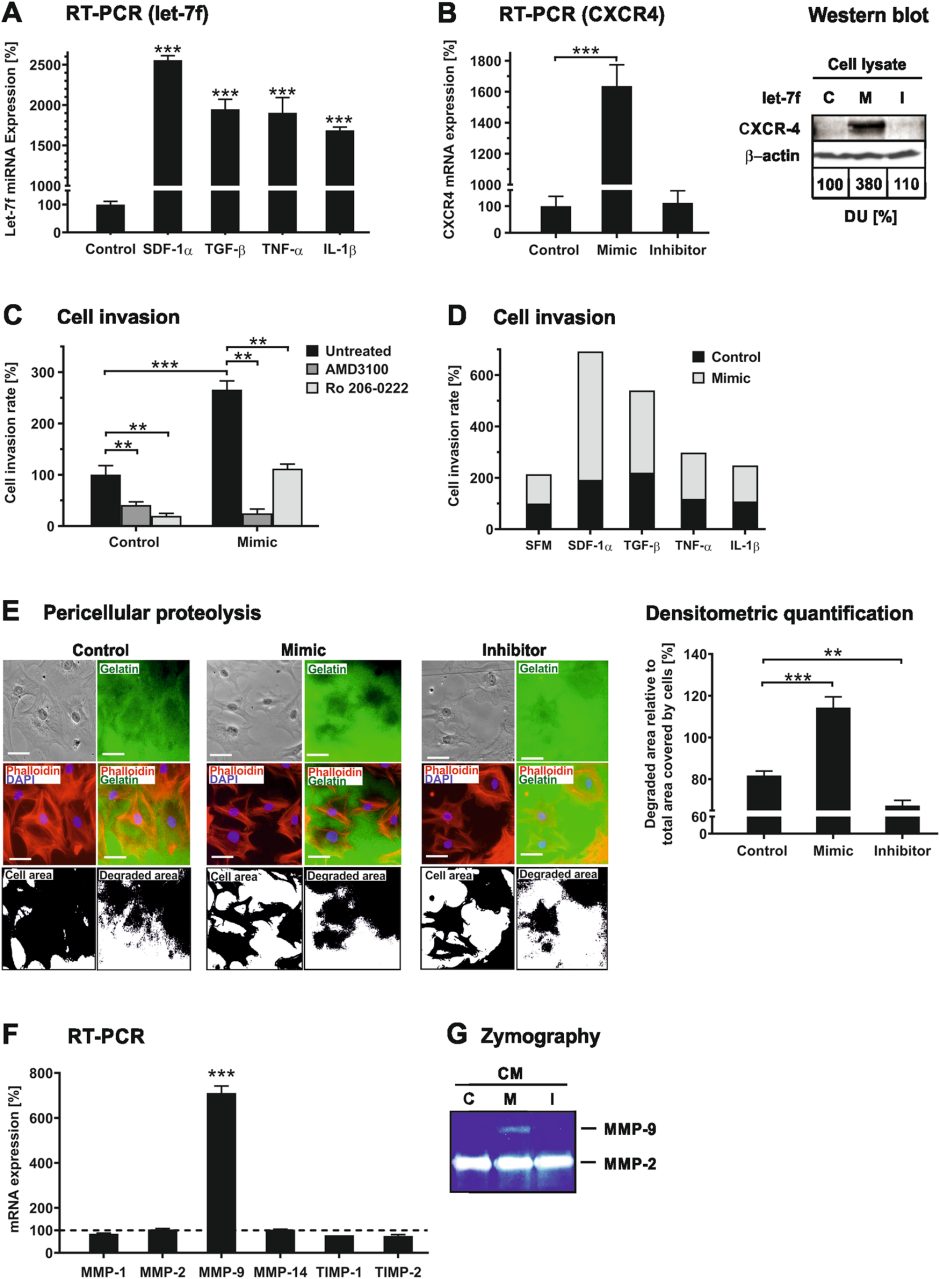
Let-7f promotes cytokine/chemokine-directed invasion of hMSCs. **A** hMSCs were incubated with SDF-1α (100 ng/mL), TGF-β1 (100 ng/mL), TNF-α (50 ng/mL), IL-1β (50 ng/mL), or left untreated (control, set as 100%) and cultivated under serum-free conditions. After 24 hours, miRNA expression of let-7f was quantified by qRT-PCR. The values shown were normalized to snoRNA. **B** hMSCs transfected with synthetic let-7f miRNA (mimic, M), antisense oligonucleotide specifically blocking let-7f activity (let-7f inhibitor, I), or non-specific oligonucleotides (control, **C** were cultivated for 24 h. Cells were analyzed for CXCR4 mRNA expression by qRT-PCR. The values were normalized to GAPDH and are given in percent to control set as 100%. Western blotting analysis of CXCR4 protein expression was performed by use of monoclonal antibodies against CXCR4. For densitometric quantification, protein from control cells was set as 100% densitometric units (DU). Intracellular β-actin was detected as loading control. **C**, **D** hMSCs were transfected with let-7f (mimic) or non-specific oligonucleotides (control). After 24 h, the cells were placed onto Transwell filters coated with human ECM and incubated for 48 h in the absence (untreated) or presence of AMD3100 or the gelatinase-specific MMP inhibitor Ro-206-0222 (each 10 µg/mL). For chemoattraction, the lower compartments of the Transwell chambers contained 10% human serum (**C**) or SDF-1α (100 ng/mL), TGF-β (100 ng/mL), TNF-α (50 ng/mL), or IL-1β (50 ng/mL). Serum-free medium (SFM) was used for the determination of spontaneous cell migration (**D**). After 48 h, the cells that had migrated into the lower compartment were counted. Control cells were set as 100%. **E** For quantitative analysis of pericellular proteolysis, the bottoms of eight-well glass chambers were coated with gelatin labeled with fluorescein. hMSCs, 24 h after transfection with let-7f (mimic), let-7f inhibitor, or non-specific oligonucleotides (control), were seeded onto the gelatin-fluorescein matrices. After 24 h of incubation, fluorescence microscopy analysis was performed for the assessment of non-degraded (green) vs. degraded areas of gelatin (no fluorescence) upon counterstaining with phalloidin-TRITC conjugate (red) and DAPI (blue) for imaging of cellular actin filaments and nuclei, respectively. The area covered by cells was recorded by phase-contrast microscopy connected to a digital camera. Scale bars indicate 5 µm. The extent of gelatin degradation in percent to the area covered by cells (set as 100%) was calculated by adopting densitometric analysis of digitally inverted pictures. **F**, **G** hMSCs were transfected with let-7f mimics (M), let-7f inhibitor (I), or control oligonucleotides (C) and incubated for up to 72 h. **F** mRNA expression of MMP-1, MMP-2, MMP-9, MMP-14, TIMP-1, and TIMP-2 was quantified by qRT-PCR after 24 h of incubation. Results are given in percentage of change in mRNA expression relative to control cells set as 100% upon normalization to GAPDH mRNA. **G** Gelatinase secretion from the cells was determined by zymographic analysis of 20 µL-aliquots of conditioned media (CM) obtained from cells cultivated for 72 h under serum-free conditions. Data shown in (**A**), (**B**), (E), and (**F**) are given as mean values ± SD of triplicate measurements (*n* = *3*). Values in (**C**) are presented as mean ± SD of one triplicate experiment representative of three independent measurements (*n* = 3). Data in (**D**) are mean values of two independent experiments (*n* = 2). **P < 0.01; ***P < 0.001.

hMSC migration is controlled by their response to various inflammatory cytokines and chemokines. First, we analyzed the effect of relevant cytokines/chemokines on the expression of let-7f in hMSCs. The inflammatory cytokines TGF-β, TNF-α, and IL-1β led to almost 20-fold higher transcription of miRNA let-7f in hMSCs, treatment with the chemokine SDF-1α to a more than 25-fold upregulation in comparison to untreated cells (Fig. 1A). CXCR4 is the specific cell surface receptor of SDF-1α^32^. Overexpression of let-7f in hMSCs by transfection with synthetic let-7f oligonucleotides (mimics) caused induction of CXCR4 mRNA levels (~16-fold) and protein levels (~4-fold) compared to cells transfected with a non-specific oligonucleotide control or an antisense oligonucleotide specifically blocking let-7f activity (let-7f inhibitor) (Fig. 1B). Tissue invasion requires the ability of cells to migrate through barriers of the ECM. Therefore, we tested the role of let-7f in this process. Overexpression of let-7f in hMSCs significantly augmented the invasive capabilities of these cells, as demonstrated by the use of Transwell cell invasion assay analysis (Fig. 1C) without affecting cell vitality (Fig. S2). In the presence of AMD3100, a specific antagonist for the binding of SDF-1α to CXCR4^33^, both basal and let-7f mimic-stimulated invasion was lower (Fig. 1C). Similarly, the addition of Ro-206-0222, a highly specific inhibitor of MMP-2, MMP-9, and MMP-14^34^, significantly reduced the invasive capacities of normal and let-7f-overexpressing hMSCs (Fig. 1C). Furthermore, we performed cell invasion assays adding SDF-1α, TGF-β, TNF-α, or IL-1β as an exclusive chemoattractant and SFM as controls into the lower compartment of the Transwell chamber. hMSCs overexpressing let-7f exhibited markedly lower random and directed cell trafficking towards the tested chemoattractants in comparison to cells transfected with control oligos (Fig. 1D).

For quantification of pericellular proteolytic activity, hMSCs were grown on a matrix of fluorescently labeled gelatin and subjected to microscopical analysis. The area of degraded gelatin was significantly larger in hMSCs transfected with let-7f mimics when compared to cells transfected with control oligonucleotides (Fig. 1E). Decreasing endogenous let-7f levels in hMSCs by transfecting the cells with a let-7f inhibitor notably reduced gelatin degradation in comparison to control cells (Fig. 1E). Interestingly, hMSCs transfected with let-7f mimics exhibited higher MMP-9 mRNA levels and protein secretion compared to control cells as determined by qRT-PCR and zymography, respectively. In contrast, no effect was observed in MMP-1, MMP-2, MMP-14, TIMP-1, and TIMP-2 mRNA levels in hMSCs upon transfection with let-7f mimics compared to control (Fig. 1F, G).

Together, these data indicate that inflammatory cytokines and the chemokine SDF-1α upregulate let-7f levels in hMSCs resulting in increased invasion and pericellular proteolysis possibly by MMP-9 secretion.

### Impact of HIF-1α and hypoxia on let-7f-promoted cell invasion

**Fig. 2:**
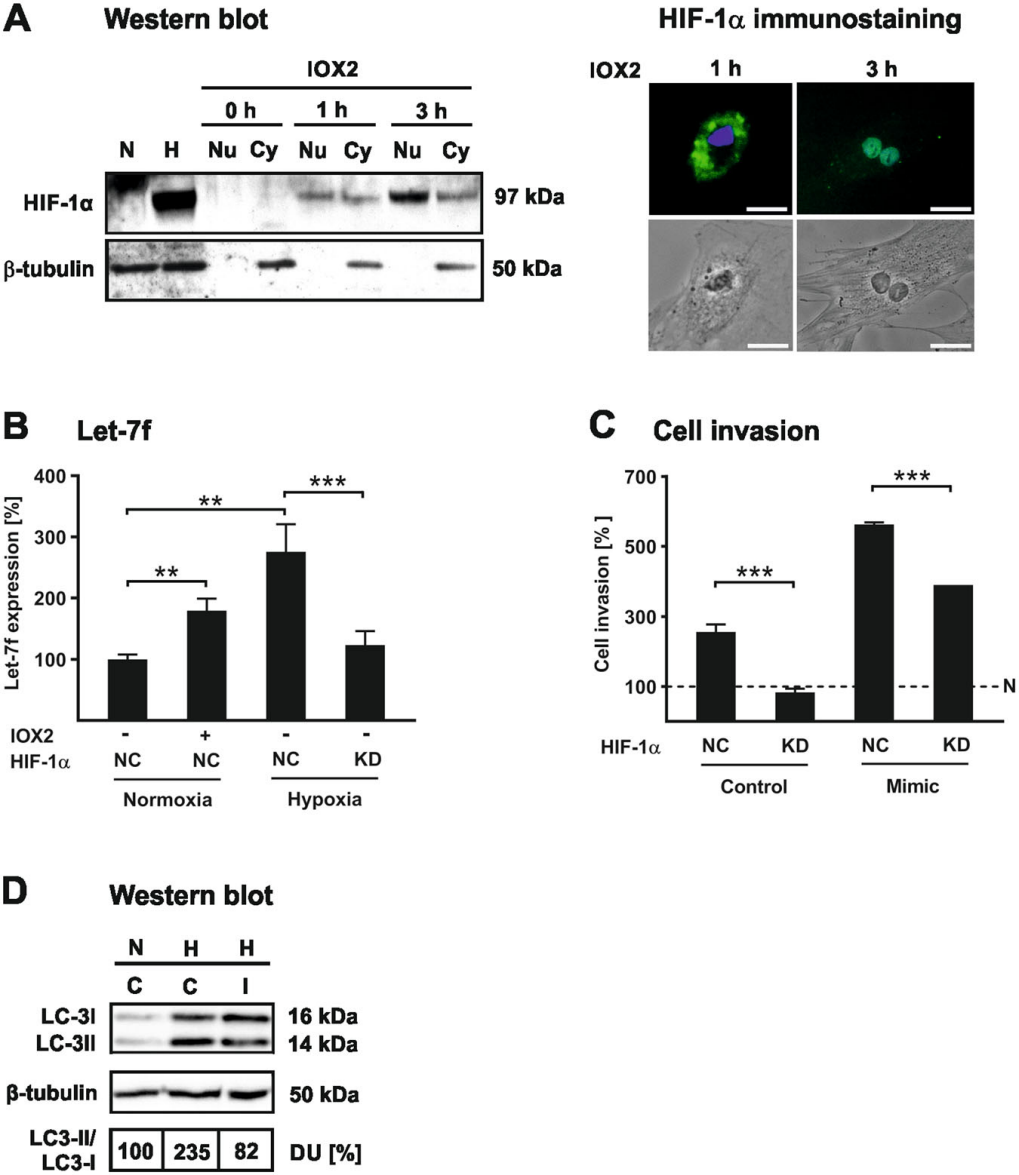
Hypoxia/HIF-1α promote cell invasion and engage autophagy through let-7f. **A** hMSCs were cultivated for 24 h under normoxic (21% O_2_) (N) and hypoxic (1% O_2_) (H) conditions, or incubated for 0, 1, and 3 h with a PHD-2 inhibitor, IOX2 (10 µM). HIF-1α levels were analyzed in whole-cell extracts (N, H), and in nuclear (Nu) and cytosolic (Cy) cell fractions by Western blotting with cytosolic β-tubulin as loading control. Subcellular localization of HIF-1α was examined by immunocytochemistry analysis after incubation of hMSCs with IOX2 (10 µM) for 1 and 3 h. Scale bars 5 µm. **B** hMSCs were transfected with HIF-1α siRNA (KD) or siRNA negative control (NC) and incubated at normoxic (21% O_2_) (N) or hypoxic (1% O_2_) (H) conditions in the absence or presence of IOX2 (10 µM). After 24 h, RNA was collected and subjected to quantification of let-7f expression using qRT-PCR analysis. The miRNA levels were normalized to snoRNA. The data represent the mean ± SD of triplicate experiments (*n* = 3); **P < 0.01, ***P < 0.001. **C** hMSCs were transfected with let-7f mimics (M), HIF-1α siRNA (KD), or siRNA control (NC) and then analyzed in the Transwell invasion assay for their potential to migrate through a barrier of human ECM toward 10% human serum as chemoattractant at hypoxic (1% O_2_) (H) or normoxic (21% O_2_) (N) conditions set as 100%. Results are presented as mean ± SD of triplicate measurements (*n* = 3) and representative for three independent experiments; ***P < 0.001. **D** hMSCs were transfected with let-7f inhibitor (I) or control oligonucleotides (C) and cultivated under normoxia (21% O_2_) (N) or hypoxia (1% O_2_) (H). After 24 h, cell extracts were harvested and subjected to Western blot analysis of LC-3I and LC3-II with β-tubulin as a loading control. The LC3II/LC-3I ratio was calculated upon normalization against β-tubulin by densitometric quantification of protein bands; control cells (C) grown under normoxia (N) were set as 100% densitometric units (DU).

Inflammation and cancer is frequently associated with hypoxia-mediated activation of the HIF-1α signaling pathway. Therefore we examined the effect of increased HIF-1α stability on hMSC invasion. When hMSCs were grown under normoxic conditions (21% O_2_), HIF-1α protein was undetectable in the cells (Fig. 2A). Under hypoxic conditions (1% O_2_) or with IOX2 treatment, a selective inhibitor of PHD2, HIF-1α protein levels were higher compared to control-treated cells (Fig. 2A). Importantly, this was accompanied by higher nuclear HIF-1α protein levels, as determined by Western blotting and immunocytochemistry analysis (Fig. 2A). Moreover, intracellular levels of let-7f were higher upon incubation with hypoxia or IOX2 (Fig. 2B). This was dependent on HIF-1α as its knockdown by siRNA-based RNAi (Fig. S3) lowered the let-7f levels back to normoxic levels (Fig. 2B). Functional analysis by use of the Transwell cell invasion assay revealed that hypoxia significantly promoted the invasive capabilities of hMSCs (Fig. 2C). This effect was augmented upon overexpression of let-7f in cells grown under hypoxic conditions, but attenuated in HIF-1α-deficient hMSCs (Fig. 2C). Interestingly, hypoxia increased the autophagic activity in hMSCs as indicated by elevated LC3-II/LC3-I ratios in comparison to cells grown under normoxia (Fig. 2D). This effect was diminished upon transfection of the cells with let-7f inhibitor (Fig. 2D).

These findings suggest that hypoxia-mediated stabilization of HIF-1α in hMSCs facilitates invasion through upregulation of endogenous let-7f in these cells associated with an activation of the autophagic pathway.

### Role of autophagy in let-7f-promoted cell invasion

**Fig. 3:**
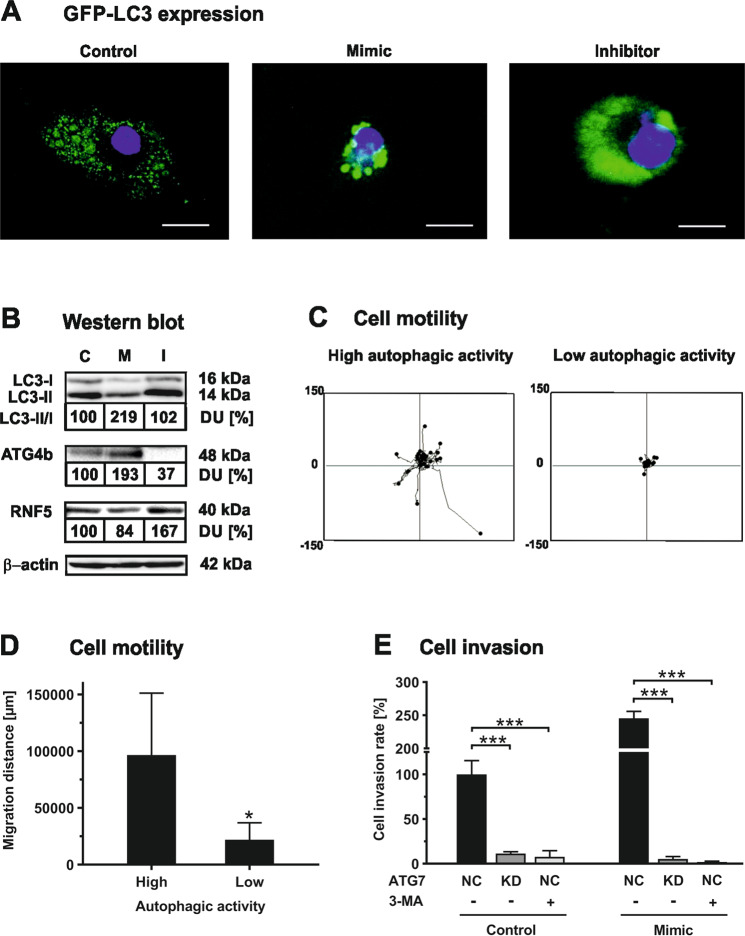
Let-7f facilitates cell invasion involving activation of autophagy. **A** hMSCs were co-transfected with a GFP-hLC3 plasmid (green) and let-7f mimic, inhibitor or non-specific control oligonucleotides. After 24 h of incubation under serum-free conditions to induce autophagy in the cells in the presence of bafilomycin A1 to inhibit autophagosome depletion, cells were examined by fluorescence microscopy analysis. Scale bars 5 µm. **B** hMSCs were transfected with let-7f mimics (M), inhibitor (I) or control oligonucleotides (C). After 24 h of cultivation under serum-free conditions, cell extracts were harvested and subjected to Western blot analysis of LC-3I, LC3-II, ATG4b, RNF5, and β-actin used as a loading control. The quantification of protein bands and LC3II/LC-3I ratio was performed upon normalization against β-tubulin by densitometry; control cells were set as 100% densitometric units (DU). The results shown are representative for four independent experiments (*n* = 4). **C**, **D** Migration paths of hMSCs transfected with GFP-LC3 plasmid and cultivated for 24 h under serum-free conditions as analyzed by fluorescence microscopy and use of the Ibidi µ-slide Chemotaxis^3D^ assay with 100 ng/mL SDF-1α as chemoattractant. The accumulated migration distance (µm) of 30 individual cells was determined adopting the Ibidi Chemotaxis and Migration Software Tool. GFP-LC3 positive cells with ≥20 puncta were classified as cells with high autophagic activity and those with less or rather diffuse staining were classified as low autophagic activity. Data shown represent the mean ± SD of triplicate measurements (*n* = 3); *P < 0.05. **E** hMSCs were co-transfected with siRNA directed against ATG7 (KD) or non-specific siRNA control (NC) and let-7f mimics or control oligonucleotides, or treated without and with an inhibitor of the autophagic pathway, 3-MA (5 µM), and studied for their invasive potential using the Transwell assay with human ECM as a barrier and 10% human serum as chemoattractant. Results are mean values ± SD of triplicate measurements and representative for three independent experiments (*n* = 3); ***P < 0.001.

Next, we studied the involvement of autophagy in let-7f-dependent hMSC invasion in more detail. To examine autophagosome formation in hMSCs by fluorescence microscopy analysis, cells were transfected with GFP-labeled LC3. Activation of the autophagic pathway in the cells was accomplished by growth in the absence of serum and the presence of bafilomycin A1, an inhibitor of autophagosome degradation. Fluorescence microscopy analysis revealed that the appearance and size of LC3-positive puncta in the cytoplasm was augmented in LC3-GFP-hMSCs upon overexpression of let-7f compared to cells transfected with non-specific oligonucleotides (Fig. 3A). In contrast, transfection of LC3-GFP-hMSCs with let-7f inhibitor resulted in a non-punctate but rather even distribution of LC3-GFP staining in the cytoplasm compared to control (Fig. 3A). These data suggested that let-7f might promote the formation of autophagosomes in hMSCs whereas let-7f deficiency impaired this process.

To confirm these observations, a detailed Western blot analysis of autophagic marker proteins was performed. Because LC3-II levels in cells show a good correlation with the number of autophagosomes present in these cells, the ratio of LC3-II to LC3-I is a useful measure of the autophagic degradation activity in cells, also called autophagic flux^35^. Transfection of hMSCs with let-7f mimics and cultivation under serum-free conditions resulted in an increase of the LC3-II/LC3-I ratio by about twofold in comparison to cells transfected with non-specific oligonucleotide controls or let-7f inhibitor (Fig. 3B). Consistently, biosynthesis of a positive regulator of autophagy, ATG4b, a proteinase that converts pro-LC3 to LC3-I, was elevated in hMSCs overexpressing let-7f when compared to control cells, whereas protein expression of the endogenous ATG4 inhibitor RNF5 was diminished (Fig. 3B). Conversely, transfecting hMSCs with let-7f inhibitor caused a decrease of ATG4b and an increase of RNF5 levels in comparison to control cells (Fig. 3B). These findings indicate that let-7f is a positive regulator of the autophagic flux in hMSCs.

To assess the relevance of autophagy in hMSC motility, we deployed life cell imaging by use of the µ-slide chemotaxis assay. LC3-GFP-hMSCs grown under serum deprivation to stimulate the autophagic pathway were tracked for 24 h and then classified into two groups according to the mainly punctate or diffuse distribution of intracellular LC3-GFP staining corresponding to high or low autophagic activity, respectively (Fig. 3C). Evaluation of the tracking analysis revealed that hMSCs with predominantly punctate LC3-GFP staining showed greater motility compared to cells with mostly diffuse staining for LC3-GFP (Fig. 3D).

Next, we studied whether autophagy had an impact on hMSC invasion. The knockdown of ATG7, an essential regulator of autophagosome assembly, or the addition of a synthetic inhibitor of autophagy, 3-MA, consistently abolished both basal and let-7f-promoted cell migration across barriers of human ECM, as determined by Transwell cell invasion assay analysis (Fig. 3E).

Together, these findings provide evidence that let-7f facilitates hMSC motility and invasion through activation of the autophagic pathway in these cells.

### Release of let-7f from hMSCs

**Fig. 4:**
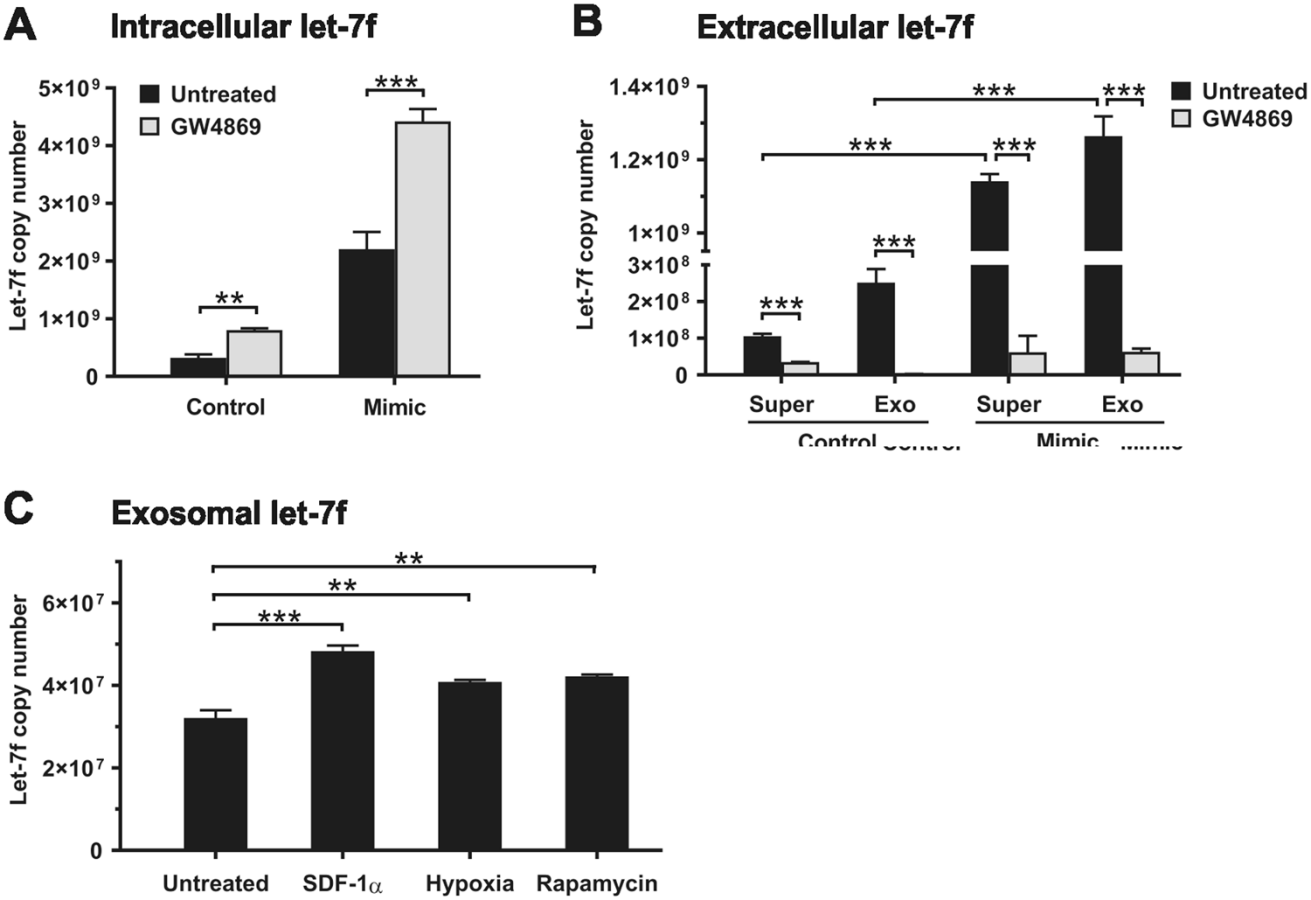
Let-7f, SDF-1α, hypoxia, and autophagy stimulate the exosomal release of let-7f. **A**–**C** hMSCs were transfected with let-7f mimics or control oligonucleotides and/or incubated for 24 h in the absence (untreated) or presence of an inhibitor of exosome formation, GW4869 (10 µM), or SDF-1α (100 ng/mL), hypoxia (1% O_2_), and an inducer of autophagy, rapamycin (5 µM). qRT-PCR analysis of absolute let-7f expression in hMSCs upon normalization against snoRNA. **A** and in culture supernatants (Super) as well as exosomes (Exo) isolated thereof (**B**, **C**). **A**–**C** Results are given as mean ± SD of triplicate experiments (*n* = 3); ***P < 0.001, **P < 0.01.

hMSCs secrete various types of vesicles that contain miRNAs. We tested the hypothesis that hMSCs release let-7f as content of exosomes. When hMSCs were cultivated in the presence of GW4869, an inhibitor of exosome secretion, the intracellular levels of let-7f were significantly increased in comparison to untreated control cells, as determined by qRT-PCR (Fig. 4A). Similarly, the addition of GW4869 to hMSCs overexpressing let-7f mimics caused a doubling of intracellular let-7f quantities in these cells compared to untreated cells (Fig. 4A).

Extracellular levels of let-7f assessed in whole culture supernatants and exosomes isolated from culture supernatants were considerably elevated upon transfection of hMSCs with let-7f mimics in comparison to cells transfected with non-specific oligonucleotide controls (Fig. 4B). Upon blockage of exosome release by treatment of the cells with GW4869, both basal and mimic-stimulated secretion of let-7f was abrogated (Fig. 4B).

Next, we were interested whether chemoattractant, hypoxia, and increased autophagy in the cells had an influence on the exosomal release of let-7f from hMSCs. Incubation of hMSCs with SDF-1α, oxygen deficiency, or rapamycin, a common inducer of autophagy, resulted in a significant augmentation of let-7f levels in exosomes isolated from culture supernatants in comparison to untreated control cells, as determined by qRT-PCR (Fig. 4C).

Taken together, our data indicate constitutive exosomal release of let-7f in hMSCs that is augmented in response to SDF-1α, hypoxia, and increased autophagy in the cells.

### Effects of hMSC-derived let-7f on tumor cells

**Fig. 5:**
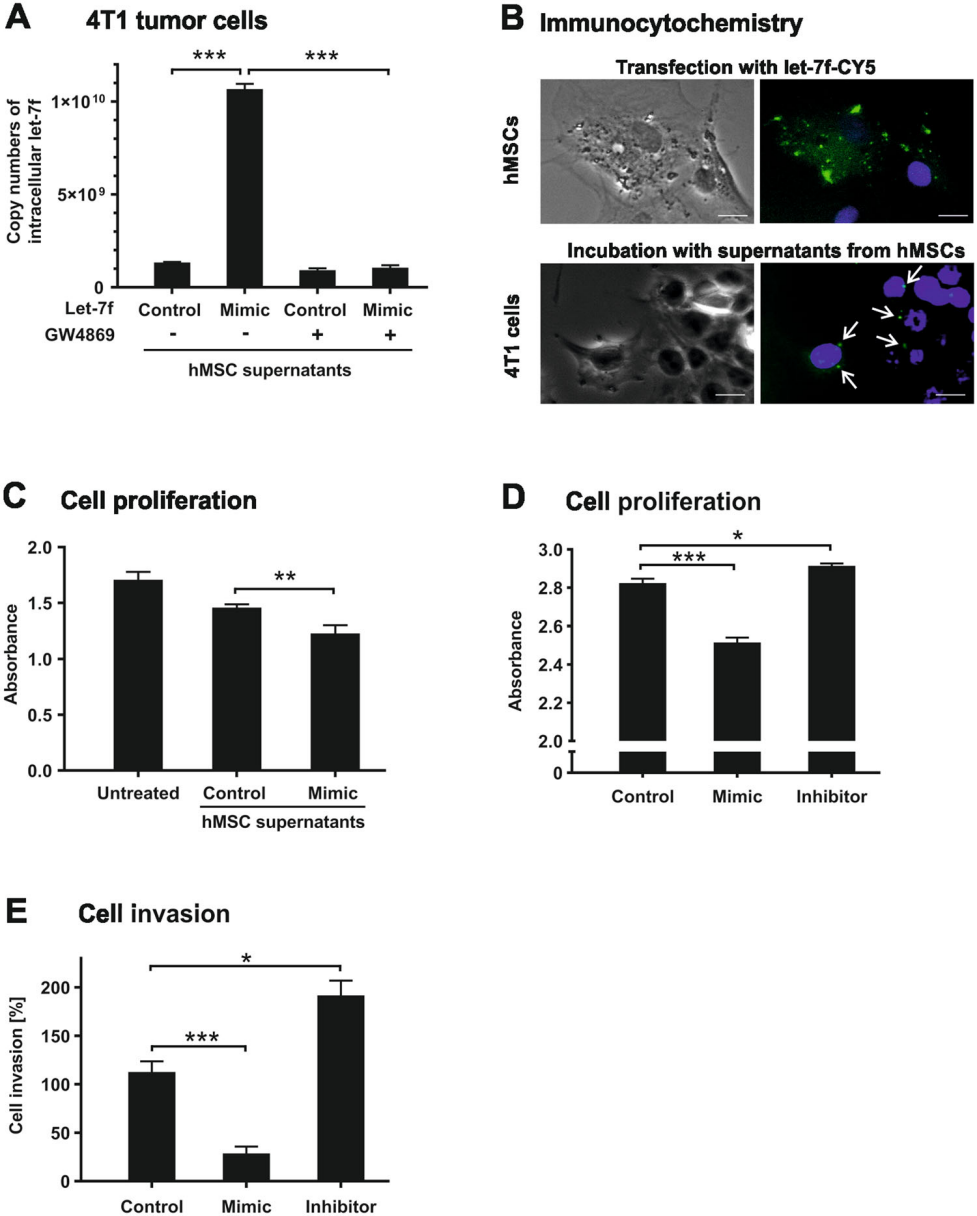
hMSC-derived let-7f is incorporated by 4T1 tumor cells impairing proliferation and invasion. **A** qRT-PCR analysis of absolute let-7f expression in 4T1 mammary carcinoma cells after 24 h of cultivation under serum-free conditions without (control) and upon addition of 24 h-cell culture supernatants obtained from hMSCs that had been transfected with let-7f mimics or control oligonucleotides grown in the absence (−) and presence (+) of an inhibitor of exosome formation, GW4869. **B** hMSCs were transfected with Cy5-labeled let-7f and analyzed by fluorescence microscopy after nuclear staining with DAPI. Scale bars 5 µm. After 48 h of cultivation under serum-free conditions, hMSC culture supernatants were collected and added to subconfluent 4T1 cells. After 24 h of incubation, 4T1 cells were analyzed for Cy5-let-7f uptake (white arrows) adopting fluorescence microscopy. **C** Assessment of cell proliferation rates in 4T1 cells upon 72 h of incubation without (untreated) and with 24 h-cell culture supernatants obtained from hMSCs transfected with let-7f mimic or control oligonucleotides by use of the WST-8 assay. **D**, **E** 4T1 cells were transfected with let-7f mimics, let-7f inhibitor, or control oligonucleotides. **D** After 72 h of incubation, cell proliferation was determined by use of the WST-8 assay. **E** Cells were analyzed for their invasive potential using the Transwell assay with human ECM as a migration barrier and 10% human serum as chemoattractant. Results are given relative to control cells set as 100%. **A**, **C**, **D** Data represent the mean ± SD of triplicate measurements (*n* = 3); ***P < 0.001, **P < 0.01, *P < 0.05. **B**, **E** Results are mean values ± SD of a triplicate measurement and representative for three independent experiments (*n* = 3); ***P < 0.001, *P < 0.05.

Exosomes play important roles in cell-to-cell communication. To test the hypothesis that let-7f released from hMSCs may be incorporated by tumor cells, we examined intracellular levels of let-7f in 4T1 mammary carcinoma cells after incubation with culture supernatants obtained from hMSCs. As determined by qRT-PCR, intracellular let-7f in 4T1 cells was significantly elevated upon exposure to conditioned media from hMSCs that had been transfected with let-7f mimics in comparison to supernatants from control hMSCs transfected with non-specific oligonucleotides (Fig. 5A). This effect was absent in 4T1 cells when incubated with supernatants from let-7f-overexpressing hMSCs treated with GW4869, an inhibitor of exosome secretion (Fig. 5A). To approve a potential uptake of hMSC-derived let-7f by 4T1 cells, we adopted fluorescence microscopy analysis. hMSCs were transfected with fluorescence-tagged let-7f (Fig. 5B). After the cultivation of the cells, the supernatants were collected and added to 4T1 cells. Subsequent microscopic examination of the 4T1 cells revealed the appearance of fluorescent puncta in the cytoplasm (Fig. 5B). These data indicate that 4T1 cells incorporate exogenous let-7f from hMSC supernatants.

We next investigated the potential influence of exogenous let-7f on 4T1 cell functions. Cells were incubated without and with supernatants from hMSCs transfected with let-7f mimics or control oligonucleotides. The proliferative activity of 4T1 cells was diminished with a significantly stronger effect obtained by supernatants from hMSCs overexpressing let-7f compared to those from control hMSCs, as determined by WST-8 assay analysis (Fig. 5C). Consistently, the direct transfection of 4T1 cells with let-7f mimics reduced cell proliferation whereas 4T1 cells transfected with let-7f inhibitor showed a slight increase (Fig. 5D).

Furthermore, we studied the impact of let-7f on 4T1 tumor cell invasion applying the Transwell invasion assay. Transfection of 4T1 cells with let-7f mimics caused a massive decline in their ability for invasion through human ECM in comparison to cells transfected with control oligonucleotides (Fig. 5E). Conversely, 4T1 cells overexpressing let-7f inhibitor showed a strong increase of invasive capacities compared to cells transfected with controls (Fig. 5E).

Together, these findings suggest that hMSC-derived let-7f is incorporated by 4T1 tumor cells and impairs proliferation and invasion of the cells.

### Influence of hMSC-derived let-7f on tumor cell growth in vivo

**Fig. 6:**
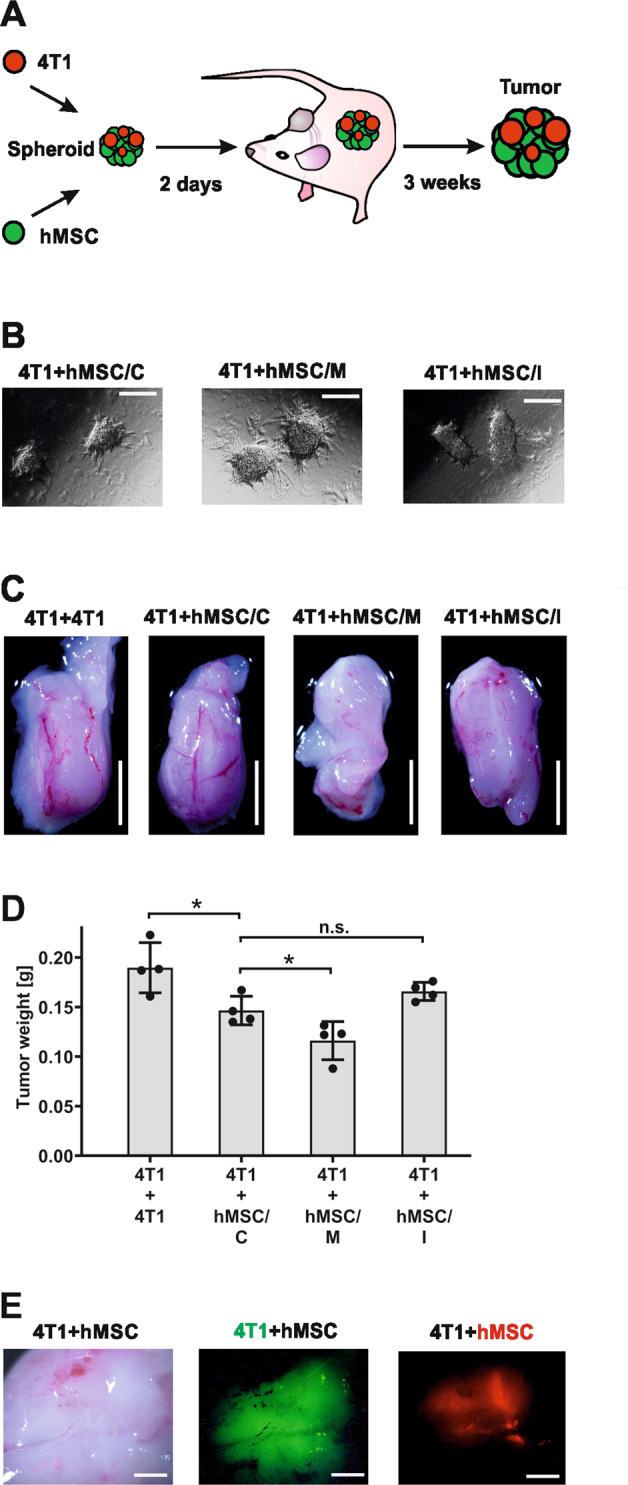
hMSC-derived let-7f has anti-tumor effect in vivo. **A** Schematic presentation of the experimental procedure and time line for use of the multicellular tumor spheroid model. 1 × 10^6^ 4T1 tumor cells were mixed in a 1:1 ratio with hMSCs transfected with let-7f mimics (hMSC/M), let-7f inhibitor (hMSC/I) or control oligonucleotides (hMSC/C) and cultivated for 2 days in collagen I gels. 2 × 10^6^ 4T1 tumor cells were used as a control (4T1 + 4T1). The resulting 3D tumor spheroids were then implanted into the inferior mammary of 16 BALB/c mice (4 mice per group). After 3 weeks, the mice were sacrificed and the tumor spheroids removed by surgery for further analysis. **B** Phase-contrast microscopy pictures representative of the resulting spheroids. Scale bars 100 µm. **C** Photographs of the respective tumors after removal from the mice. Scale bars 5 mm. **D** The results of quantification of the tumor masses determined by weighing are shown. **P < 0.01, n.s. not significant. **E** 4T1 cells stained with CellTracker CMFDA (green fluorescence) and hMSCs stained with CellTracker CMTPX (red fluorescence) were mixed in a 1:1 ratio. The resulting spheroids were implanted into the inferior mammary of BALB/c mice and after three weeks analyzed by intravital microscopy analysis. Photographs of the respective tumor after removal from the mice. Scale bars 1 mm.

To provide in vivo evidence for the relevance of hMSC-derived let-7f, we adopted a three-dimensional mouse tumor model^36^ (Fig. 6A). To study the crosstalk of hMSCs with 4T1 tumor cells, spheroids were generated consisting of 4T1 cells and 1:1 mixtures of 4T1 cells and hMSCs that had been transfected with let-7f mimic, let-7f inhibitor or control oligonucleotides (Fig. 6B). Three weeks after implantation of the spheroids into the inferior mammary of BALB/c mice, the resulting tumor tissue was harvested and weighted. Both visual evaluation by microscopy (Fig. 6C) and tissue mass assessment (Fig. 6D) revealed that the tumors harboring hMSCs were smaller in size compared to those grown from exclusively 4T1 cells. Importantly, the mass of tumors grown from 4T1 cells mixed with hMSCs overexpressing let-7f was significantly less than in tumors of 4T1 cells and hMSCs transfected with non-specific oligonucleotides (Fig. 6C, D). In contrast, tumors that had developed from 4T1 cells mixed with hMSCs overexpressing let-7f inhibitor were slightly larger in size and weight compared to tumors obtained from 4T1 cells combined with hMSCs transfected with control oligonucleotides (Fig. 6C, D). CellTracker staining analysis revealed that the tumors isolated three weeks after mixed spheroid implantation harbored a substantial proportion of live hMSCs (Fig. 6E). Taken together, our data indicate the relevance of let-7f in hMSC-mediated anti-tumor effects in vivo.

## Discussion

hMSCs are characterized by their tumor tropism and property of chemotactic migration to inflamed tissues. Our published work has shown that inflammatory cytokines and SDF-1α facilitate hMSC invasion by increased MMP production by the cells^2^ but the underlying regulatory mechanism was unknown. Here we provide evidence that let-7f is a key regulator of hMSC invasion in response to inflammatory cytokines and SDF-1α previously shown to control MMP expression in these cells^2^. Overexpression of let-7f in hMSCs upregulated the SDF-1α receptor CXCR4, elevated the release of ECM-degrading MMP-9^37^, and augmented the level of pericellular proteolysis by the cells. Under physiological conditions, this mechanism may contribute to hMSC recruitment in response to tissue injuries and inflammation. On a molecular basis, let-7f is likely to target repressors of cellular susceptibility and chemotactic migration thereby acting as promoter of hMSC invasion. Remarkably, an opposite role has been reported on several let-7 family members in various cancers operating as tumor suppressors by inhibiting growth and MMP-dependent invasion of malignant cells^38–40^.

Hypoxia is a hallmark of myocardial ischemia and cancer and is known to recruit hMSCs^9,41,42^. Interestingly, preconditioning of MSCs with hypoxia was shown to improve migration, survival, and immunosuppressive effects by these cells in vivo^43–45^. Our study reveals that hypoxia/HIF-1α-stimulated hMSC migration is regulated through let-7f which has been shown to belong to the hypoxia-responsive family of miRNAs^46^. The effect Let-7b has a similar role downstream of HIF-1α in hypoxia-mediated cell proliferation and cell cycle regulation in zebrafish^47^. Moreover, we show hypoxia/HIF-1α to stimulate the autophagic pathway in hMSCs. Autophagy is integral to the well-orchestrated programs of cellular responses to stress and exhibits a cytoprotective role that is balanced by apoptosis having a cytodestructive function^48^. Our study elucidates a mechanism by which let-7f triggers autophagy through opposite effects on an executor (ATG4b) and an inhibitor (RNF5) of autophagic activity resulting in enhanced hMSC invasion. This is consistent with previous findings in neural cells showing that let-7 controls the migration of these cells through positive regulation of autophagy involving the mTOR signaling pathway^49,50^. Notably, MSCs engineered to overexpress let-7f exhibited improved in vivo survival by impaired apoptosis through let-7f targeting of endogenous caspase-3^24^. Given the opposing role of autophagy and apoptosis, it appears conceivable that let-7f is a key regulator of the crosstalk between both processes. In summary, let-7f is central in the control of hMSC proliferation, differentiation, and migration emphasizing its relevance in MSC-based therapies^17^.

The secretome of hMSCs contains numerous bioactive factors attributed to exert therapeutic effects in multiple diseases^11^. Investigating the role of let-7f and its regulation in this context, we discovered a direct correlation between endogenous and extracellular levels of let-7f in hMSCs, indicating that the mechanisms regulating let-7f expression in the cells are closely linked to those controlling its release. Indeed, SDF-1α, hypoxia, as well as activation of autophagy not only augmented endogenous let-7f in hMSCs but also enhanced its secretion in exosomes. Accumulating evidence indicates that MSC-derived exosomes may provide a useful drug in paracrine anti-cancer therapies by horizontal transfer of their cargo into host cells present in tumor tissue^13,51^. Our study reveals that let-7f-enriched exosomes obtained from let-7f-overexpressing hMSCs are incorporated by breast cancer cells. Moreover, the let-7f-enriched exosomes upon uptake into tumor cells impaired proliferation and invasion more efficiently than non-let-7f-enriched exosomes. This can be explained by the fact that in cancer let-7 is well known for its tumor suppressor function due to its targeting of RAS genes essentially involved in the progression of the disease^52^. Various pathomechanisms account for significantly lowered let-7 expression levels in numerous tumors. For instance, let-7 depletion can result from a dysregulation of HIF-1α-controlled autophagy in malignant cells^53^. Consistently, the let-7 levels in breast cancer tissues are also low and overexpression of let-7 was shown to block tumor cell invasion^39,54^. This is in agreement with our findings on let-7f in 4T1 breast cancer cells supporting the concept that MSCs affect host cells largely via their paracrine factors^55^.

Cancer management using MSCs stems from the ability of these cells to home to tumors. The process of tumor tropism involves MMP secretion by MSCs and crosstalk via the SDF-1α/CXCR4 axis^56^. Our findings suggest a pivotal role of let-7f in the cytokine/chemokine-mediated tumor tropism of hMSCs involving its positive regulation of MMP-9 and CXCR4. Furthermore, using a mouse 4T1 breast tumor model we show that hMSCs overexpressing let-7f in close proximity to the cancer cells display an improved anti-tumor potential, most probably mediated via exosomal delivery of let-7f. Similar anti-proliferative effects of miRNA-containing exosomes from MSCs have been described in ovarian cancer cells^57^. However, despite their multiple anti-tumorigenic functions MSCs are also involved in tumor-promoting processes including angiogenesis and therapy resistance^58^. Hence, further efforts are warranted for the optimization of preconditioning protocols in MSC-based therapeutical applications and for the use of MSC-derived exosomes in cell-free therapies.

**Fig. 7:**
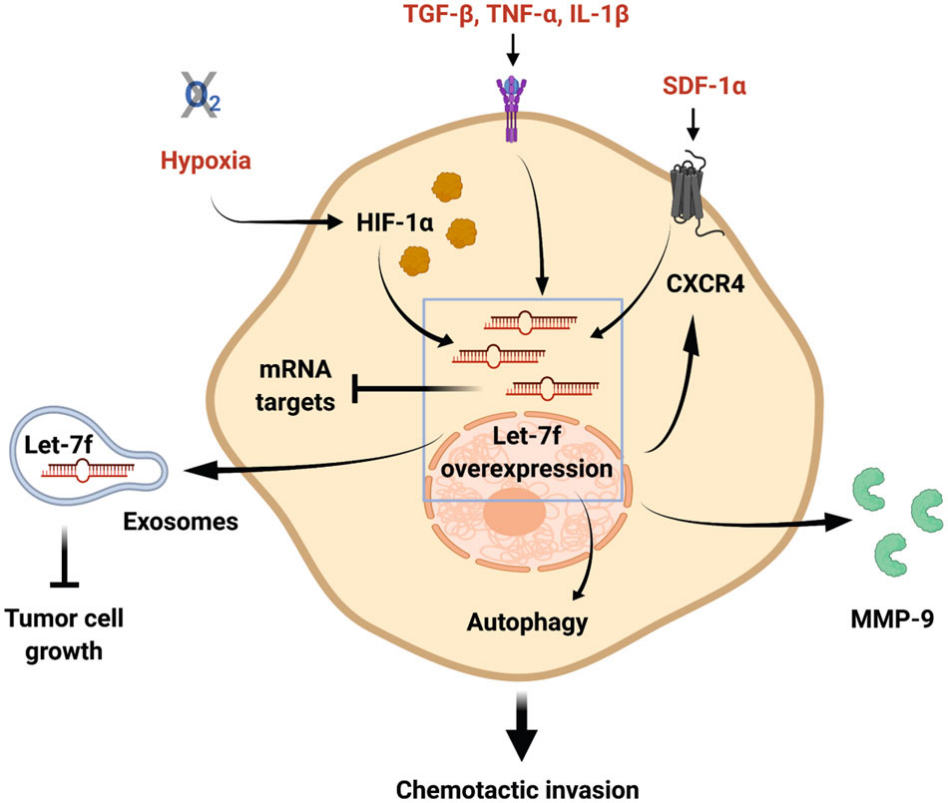
Visual summary of the main findings of the study. hMSC exposure to inflammatory cytokines, SDF-1α, and hypoxia upregulates let-7f in the cells promoting chemotactic invasion via engagement of autophagy and release of ECM-degrading proteases. In a paracrine manner, exosomal let-7f affects tumor growth.

In conclusion, we have demonstrated that let-7f is a positive regulator of hMSC invasion in response to chemotactic cytokines and hypoxia/HIF-1α by activating the autophagic pathway (Fig. 7). Furthermore, our results show that overexpression of let-7f in hMSCs creates let-7f-augmented exosomes with an improved anti-tumor effect in vivo. Additional detailed studies are required for a deeper knowledge of the regulatory network based on let-7f-controlled stem cell functions relevant in hMSC´s multifaceted roles in tissue repair and disease.

## Supplementary information

Supplementary Figures

Supplementary Tables
