# Supplementary material for: Let-7f miRNA regulates SDF-1α- and hypoxia-promoted migration of mesenchymal stem cells and attenuates mammary tumor growth upon exosomal release
Source: Cell Death Dis. 2021 May 20;12(6):516. doi: 10.1038/s41419-021-03789-3 (PMC8137693; doi:10.1038/s41419-021-03789-3)
Supplement: Supplementary file 1 — Supplementary Figures [file 41419_2021_3789_MOESM1_ESM.docx]

**Supplementary FIGURE 1**


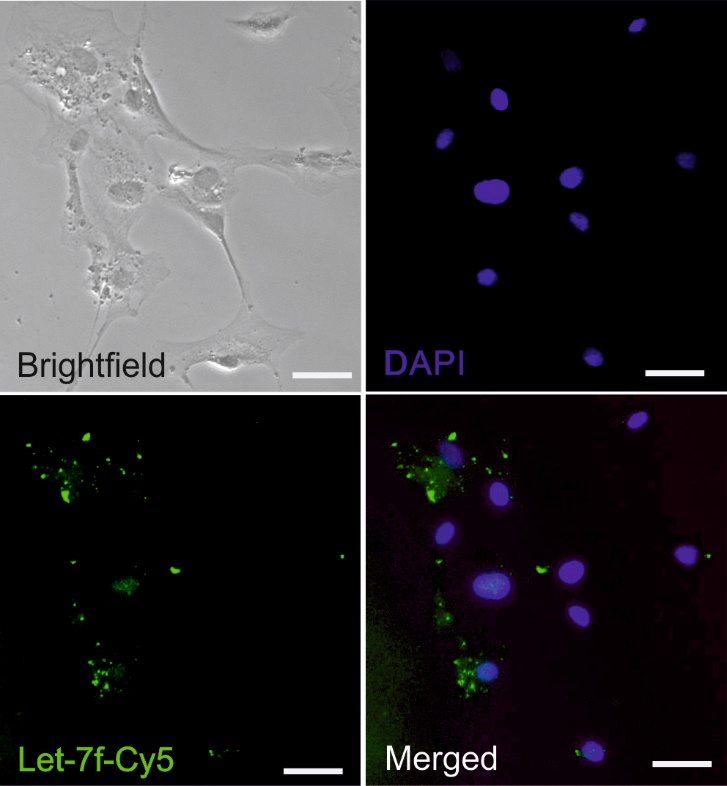


**Fig. S1. Cell transfection efficiency analysis.** hMSCs were transfected with Cy5-labeled let-7f (Let-7f-Cy5) and analyzed by brightfield and fluorescence microscopy after nuclear staining with DAPI. In the microscopic section as presented here, 7 of 9 cells show positive for uptake of let-7f-Cy5. Scale bars 5 µm. The results are representative for several independent experiments.

**Supplementary FIGURE 2**





**Fig. S2. Effect of let-7f mimic and inhibitor on hMSC vitality.** hMSCs transfected with synthetic let-7f miRNA (mimic, M), antisense oligonucleotide specifically blocking let-7f activity (let-7f inhibitor, I), or non-specific oligonucleotides (control, C, set as 100%) were cultivated for 24 h and subsequently analyzed for mitochondrial dehydrogenase activity using the WST-8 assay. The data represent the mean ± SD of triplicate experiments (*n* = 3).

# Supplementary FIGURE 3





**Fig. S3. Time dependent knockdown analysis of HIF-1α.** hMSCs were transfected with HIF-1α siRNA (KD) or siRNA negative control (NC). After 1 day and 3 days of incubation, RNA was collected and subjected to quantification of HIF-1α expression using qRT-PCR analysis. The data represent the mean ± SD of triplicate experiments (*n* = 3).
