# Supplementary material for: Let-7f miRNA regulates SDF-1α- and hypoxia-promoted migration of mesenchymal stem cells and attenuates mammary tumor growth upon exosomal release
Source: Cell Death Dis. 2021 May 20;12(6):516. doi: 10.1038/s41419-021-03789-3 (PMC8137693; doi:10.1038/s41419-021-03789-3)
Supplement: Supplementary file 2 — Supplementary Tables [file 41419_2021_3789_MOESM2_ESM.docx]

# Supplementary Tables

**Table S1.** qPCR primer sets and sequences of miRNA mimics, inhibitors and siRNAs

| **Target gene** | **Sequence Forward** | **Sequence Reverse** |
| --- | --- | --- |
| hsa_ATG7 siRNA | Qiagen cat. no. SI04194883 | |
| hsa_GAPDH | AATGAAGGGGTCATTGATGG | AAGGTGAAGGTCGGAGTCAA |
| hsa_HIF1A | TGAGGACTTGCGCTTTCAGG | CCCATTTTCTACTCAGGACAC |
| hsa_HIF1A siRNA | 5´-CAAGCTCAAGCCATACATAAA-3´ | |
| hsa_let-7f-3p | Qiagen miScript cat. no. MS00008274 | |
| hsa_let-7f-3p mimic | 5´-UGAGGUAGUAGAUUGUAUAGUU-3´ | |
| hsa_let-7f-3p inhibitor | 5´-UGAGGUAGUAGAUUGUAUAGUU-3´ | |
| hsa_MMP1 | Search-LC cat. no. 487577 | |
| hsa_MMP2 | Search-LC cat. no. 487584 | |
| hsa_MMP9 | Search-LC cat. no. 487645 | |
| hsa_MMP14 | Search-LC cat. no. 487651 | |
| hsa_TIMP1 | Search-LC cat. no. 487553 | |
| hsa_TIMP2 | Search-LC cat. no. 487560 | |
| Snord61 | Qiagen miScript cat. no. MS00033705 | |
| Snord72 | Qiagen miScript cat. no. MS00033719 | |
| U2 | CATCGCTTCTCGGCCTTTTG | TGGAGGTACTGCAATACCAGG |

Primers were purchased from Sigma-Aldrich, Search-LC, or Qiagen

**Table S2.** List of antibodies.

| **Antigen** | **Supplier** | **Clone /**  **Cat. no.** | **Concentration** | |
| --- | --- | --- | --- | --- |
|  |  |  | **WB** | **IF** |
| ATG4b | CST | D11/3868 | 1:1000 | ~~-~~ |
| CXCR4 | MBL | M-5224 | 1:1000 | - |
| HIF-1α | BDL | 54/HIF1a | 1:1000 | 1:200 |
| LC3-I | CST | D50G8/4599 | 1:1000 | - |
| LC3-II | CST | D11/3868 | 1:1000 | ~~-~~ |
| RNF5 | Abcam | Ab83466 | 1:1000 | - |
| β-actin | Abcam | ab8227 | 1:100000 | - |
| β-tubulin | Abcam | ab6072 | 1:2000 | - |
| Goat anti-mouse IgG (Star635P) | Abberior | 2-0002-007 | 1:2000 | 1:250 |

WB, Western blot; IF, immunofluorescence; BDL, BD laboratories; CST, Cell Signaling Technology.
